# To protect healthcare as an affordable and valued service, we need to segment as much of the pathway as possible into products

**DOI:** 10.1016/j.fhj.2024.100145

**Published:** 2024-05-18

**Authors:** Dr Mark Vignesha Roberts

**Affiliations:** Lead Consultant Integrated Care, Western Health and Social Care Trust, Senior Medical Officer Acute Care Policy, Department of Health Northern Ireland

## Abstract

Maintaining care quality and affordability, in the face of increasing demand, complexity and resource constraints, is becoming more and more challenging. A mindset shift towards how we approach healthcare services is needed. I propose we segment much of the healthcare pathway into products, harnessing the capabilities of software and hardware, including judicious yet ambitious application of emerging artificial intelligence capabilities. In this future, digital tools and capabilities that enhance a greater proportion of self-care and signposting will do more of the heavy lifting of healthcare, vitally freeing up healthcare staff including doctors to do the higher-value relational, technical, and leadership and assurance tasks needed in this future landscape.

It is 2043. I'm retiring from our National Health Service and one of my colleagues says a few words of kindness in front of a packed seminar room. Junior members of the team look visibly bemused when the 2020s are described as a time of organisational chaos, dwindling recruitment and retention successes, seemingly unresolvable cost pressures and accelerating demand for services. That was, she says, until the country pivoted substantially and channelled more and more money into the health service from the mid-2020s until the present time, recruiting health and social care staff of all disciplines, largely fixing key bottlenecks in care pathways and marrying this with record investments in pharmaceuticals, community and hospital infrastructure.

This future isn't going to happen for two reasons. I'm not that popular so the seminar room won't be packed. More importantly, no government in the world will commit such sustained increases in the proportion of the gross domestic product (a recognised measure of the strength of a country's economy) required from the current levels of 11–12% ^1^ to 22–25%.

Viewing healthcare as a product, not a service, invites inflamed and uncomfortable emotions in doctors and many others connected to the health service. We have been schooled on a diet of the centrality of caring for people in their hours, days and years of need and investing in the relational elements of the doctor–patient interaction in order to respond to life's complexities from the cradle to the grave. We may regard discussions around resourcing and sustainability as vulgar or unpalatable, or a description of healthcare as a series of interconnected products as demeaning or reductionist. However, I believe the model we currently operate is on a terminal trajectory, in part because it is fraying from the unsustainable weight of expectation on staff doing the heavy lifting. Moving the discourse away from ‘public vs private’ funding and towards a concept of ‘products and services’ can help.

A service relies on the heavy lifting being done by people, while a product can be produced, modified and scaled up with relative ease. Crucially, once the early costs are met, the costs per unit, interaction and outcome start tumbling. First iterations are never an overnight success. For example, when bicycles were first introduced in the 19th century, they were uncomfortable, unwieldy and expensive for all but deep-pocketed enthusiasts. They made no impact in the service of ‘mass transportation’. But as usability and affordability rocketed in the 20th century, these products made more and more impact in the world of transportation as their perceived value by users increased more and more. Now, the transport system of many major cities around the world would be paralysed were it not for the humble bicycle's contribution to mass transport every day.

The analogy with the healthcare system is that we haven't designed and built enough bicycle equivalents to keep up with demand. We have inadvertently built an industry that in effect tells people they must order an approved taxi, bus or train to get anywhere. Product and technology enabled self-care and agency are not enfranchised with products akin to a bicycle. If, as some economists might argue, products ‘are distribution mechanisms for service provision’,^2^ the presence of an estimated 1 billion bicycles in the world speaks concretely of this product's ability to deliver a useful service to an individual's transportation needs. What is the equivalent to the bicycle that we need to design for the health service?

The current direction of travel predicates on recruiting and retaining more and more staff to operate a health service which, despite its rhetoric, often fundamentally operates with the patient and citizen as passive recipients and staff carrying the overwhelming weight of service provision. We have to turn sharply away from this because the financial and human resources required are not sustainable, and because insufficient emphasis is placed on what patients and citizens value. At present the costs per unit of output in the health service rise in ever steeper curves. This is partly due to pressures from wages which increase over time incrementally, a reality I would be insincere to argue against as an employed doctor. But it is crucially also because I, as a single doctor, have only so many working hours I can devote to face-to-face or remotely managed care – I can't be scaled. When over 60% of the health service budget^3^ is spent on primary and secondary care staff wages, you can see how limiting the realities of service expansion are, when the rate-limiting step of expansion and growth is – ‘*how many people do you have*?’.

We need to judiciously, and with a huge dose of ambition, harness the combined growing power of artificial intelligence (AI), software and hardware capability. We can do this by intentionally creating the conditions where software solutions that meet citizen and patient need can flourish and catalyse elements of the healthcare pathway. Where they do so in a proportionately regulated but widely accessible space, most notably in the fields of enfranchised self-care, screening, diagnosis, treatment and aftercare. This frees existing healthcare staff to focus on delivering services in areas where higher-complexity, technical skill and/or relational needs require it. AI-driven software capability is already happening in pockets of services such as in neuroimaging analysis in acute stroke victims^4^ or triaging processes for patients with photographs of suspected skin cancers.^5^ Nonetheless, these impacts are nothing like at the scale, ambition and breadth we need to embrace.

Compared to indefinite, incremental and unsustainable increases in workforce funding to satisfy the current model, the existing workforce would instead benefit from time-limited investment and mental gear changes to enable it to play an effective partnership role with the technological capabilities. In this scenario, doctors and other healthcare professionals would act as trusted sense-checkers, navigators and deliverers of technical and non-technical high value tasks that software products alone cannot deliver on. These might include complex communication or risk assessments, operations and procedures, and team working, quality assurance and leadership skills, to name a few. By weighting effectively the voices of staff (in clinical, support and leadership roles), this will help identify signals of success and failings. Interpretation and effective response to these signals are part of the skill set that will be required for future health and social care leaders and managers. The most effective compass for directing us collectively to sustainable success will be ensuring patients and citizens have a dominant influencing role in identifying what services and experiences are of value.

We will need to accept that the next 5–10 years for healthcare and the country will be challenging but there is a very important difference to the often painful and draining space we are in now. We should be powered by the confidence that we can, with careful custodianship of a vital service for citizens, move towards a future that has abundance of expertise and quality at scale and lower cost. This is a solution where scalable technology is doing the heavy lifting and people are doing the elements we as a democratic society deem both of value and affordable.

Before my retirement in 20 years, delivering a more effective, affordable and hopeful vision for the health and social care system is within grasp. I believe we've reached the tipping point when we need to take it.

## Declaration of competing interest

The author declares that he has no known competing financial interests or personal relationships that could have appeared to influence the work reported in this paper.
